# PET/Bio-Based Terpolyester Blends with High Dimensional Thermal Stability

**DOI:** 10.3390/polym13050728

**Published:** 2021-02-27

**Authors:** Sangyoon Park, Sarinthip Thanakkasaranee, Hojun Shin, Youngsoo Lee, Guman Tak, Jongchul Seo

**Affiliations:** 1 Department of Packaging, Yonsei University, 1 Yonseidae-gil, Wonju-si, Gangwon-do 26493, Korea; sypark@splatech.com (S.P.); s.thanakkasaranee@gmail.com (S.T.); ghwns0310@naver.com (H.S.); 2 Saehanplatech Inc., 851-11, Dulleung-ri-ro, Ochang-eup, Cheongwon-gu, Cheongju-si, Chungcheongbuk-do 28017, Korea; marlee@splatech.com (Y.L.); 17014@splatech.com (G.T.)

**Keywords:** PET, PEICT, bio-based terpolyester, semi-crystalline/amorphous blend, high dimensional thermal stability

## Abstract

To improve the dimensional thermal stability of polyethylene terephthalate (PET), a poly(ethylene glycol 1,4-cyclohexane dimethylene (CHDM) isosorbide (ISB) terephthalate) (PEICT) known as ECOZEN^®^T110 (EZT) was introduced into PET using a melt blending technique. The miscibility, morphology, and thermal properties of the PET/EZT samples were investigated. The introduction of amorphous EZT into semi-crystalline PET increased the glass transition temperature (T_g_) but decreased the crystallinity, which could be related to the transesterification reaction. By adding EZT contents up to 20%, the PET/EZT samples showed a single T_g_, which indicated the miscibility between PET and EZT. However, two T_g_ values were observed in the PET/EZT samples with higher EZT contents (30–70%), indicating partial miscibility. This may have been due to the slightly different rheological and thermodynamic parameters that were affected by a higher ratio of bulky (rigid ISB and ductile CHDM) groups in EZT. However, the heat distortion temperature of the PET/EZT samples remarkably increased, which indicated that the dimensional stability was truly enhanced. Although the crystallinity of the PET/EZT samples decreased with increasing EZT content, the tensile strength and Young’s modulus decreased slightly. Based on these results, the as-prepared PET/EZT samples with high dimensional stability can be used as a high-temperature polymeric material in various applications.

## 1. Introduction

Poly(ethylene terephthalate) (PET) is a semi-crystalline thermoplastic polymer that has been widely used in the packaging, textile, and automotive industries owing to its excellent mechanical properties and chemical resistance [1]. However, PET has a low heat distortion temperature (HDT) and is limited in product design for high-temperature applications [2]. An improvement in the dimensional thermal stability of PET can be achieved by increasing the degree of crystallinity, which is commonly used in the conventional process to prepare a bottle for the hot-filling process (pasteurization) in the beverage industry (i.e., tea, fruit juices). However, highly crystalline PET bottles are typically prepared by increasing the crystallization time (cooling time) or using complex machines, leading to low productivity and high investment costs [3]. In addition, several researchers reported that the introduction of rigid nanoparticles (e.g., carbon nanotubes, nanoclays, and graphene oxide) into PET resulted in an increase in the HDT of PET nanocomposites, leading to higher dimensional stability [4,5,6]. However, the aggregation of these nanoparticles and insufficient interfacial interactions between nanoparticles and the polymer matrix are the main disadvantages of this method in increasing the HDT of the pristine polymers [1]. In addition, the aggregated nanoparticles can reduce the transparency of PET [1,7], which is limited for product/packaging designs (i.e., primary packaging). Therefore, surface modification of the nanoparticles is required prior to nanocomposite preparation [1,8], leading to an increase in production time owing to the two-step method. In addition, the application of the PET nanocomposites in food packaging may result in the migration of nanoparticles from the polymer matrix to packed foods [9].

The blending of polymers has been extensively employed to develop new polymeric materials owing to their advantages [10,11]. For instance, polymer blending is a convenient way of developing materials with newly enhanced properties by combining the excellent properties of the different existing polymers [10,11]. However, the thermodynamic immiscibility of most polymers is a considerable challenge in the development of polymeric systems [10,12,13]. In general, HDT is related to the glass transition temperature (T_g_) of the polymeric materials and the relaxation of molecular segments of the plastic materials under defined loads [11,14,15]. Therefore, an increase in T_g_ is an alternative method to enhance HDT, as it improves performance under high-temperature conditions. It is worth noting that the blending of polymers having the compatible chemical structure results in the chemical interactions (e.g., transesterification during melt blending), which may be led to the miscible products with stronger properties compared to starting components.

**Figure 1:**
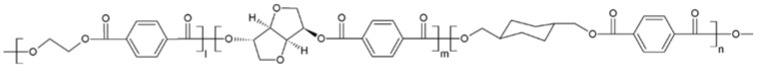
The chemical structure of the terpolyester PEICT; ethylene glycol (EG) (l), isosorbide (ISB) (m), and 1,4-cyclohexane dimethanol (CHDM) (n). Reproduced from Ref. [17] with permission from American Chemical Society.

In a previous study [16], a high heat resistance and shape stability of PET/bio-based terpolyester bottles were achieved by incorporating the poly (ethylene glycol 1,4-cyclohexane dimethylene isosorbide terephthalate) (PEICT), ECOZEN^®^HF502, via injection stretch blow molding. The PET/PEICT samples exhibited a single T_g_, which increased from 77.0 to 84.0 °C owing to the rigid molecular structure of the isosorbide (ISB) group in the PEICT. Moreover, the PET/PEICT bottle exhibited high transparency owing to a truly miscible sample, which can be applied in the hot-filled process in the beverage industry [16]. However, PEICT has different grades depending on the ratio of functional groups, and only a few studies of semi-crystalline PET/amorphous PEICT samples have been reported; therefore, investigations into the new outstanding properties of these blends are challenging. In addition, the use of bio-based polymers in polymeric systems is an alternative to reduce the use of fossil-based polymers. The chemical structure of PEICT is shown in Figure 1.

In this study, PEICT with a high ratio of ISB and 1,4-cyclohexane dimethanol (CHDM) (compared to the previous study) is known as ECOZEN^®^ T110 (EZT), was used to prepare high-performance PET/bio-based terpolyester for high-temperature applications. The changes in HDT and T_g_ of the samples and the miscibility between PET and EZT were thoroughly investigated.

## 2. Materials and Methods

### 2.1. Materials

Semi-crystalline PET, under the code name BCN 80, was provided by Lotte Chemical Co. Ltd. (Yeosoo, South Korea). The bio-based terpolyester PEICT consisting of ISB, EG, CHDM, and terephthalic acid, under the code name ECOZEN^®^ T110, was provided by SK Chemical Co. Ltd. (Ulsan, South Korea).

### 2.2. Preparation of PET/EZT Sheets

The PET and EZT resins were mixed using a tumbler mixer CTTM-200S (Crutec Co., Ltd., Siheung, South Korea) and melt blended using a twin-screw lab extruder (UNEE PLUS, Hwaseong, South Korea) to obtain homogeneous compounded samples. The barrel temperatures were set between 220 and 270 °C for zones 1 to 6 and 280 °C for the header. Prior to sheet fabrication, the compounded samples and EZT resin were dried at 85 °C for 4 h, whereas PET was dried at 150 °C for 4 h.

PET, PET/EZT compounded samples, and EZT were individually added into the MA 600e hybrid precision injection molding machine (Haitian Korea Co., Ltd., Bucheon, South Korea) to obtain the pristine PET, PET/EZT, and pristine EZT sheets, respectively. The temperature of the barrel and nozzle was set between 255 and 275 °C. The molten sample was injected into the mold. Then, the sheets were rapidly cooled in the mold to 40 °C within 30–40 s. PET, PET/EZT, and EZT sheets were finally obtained with a thickness of 1.0 ± 0.1 mm. The different ratios of the PET/EZT samples were prepared as follows: 100:0, 90:10, 80:20, 70:30, 50:50, and 30:70, in which the first and second values indicate the ratio of PET and EZT in the sample.

### 2.3. Characterizations

The FT-IR spectra of the pristine PET, PET/EZT, and pristine EZT samples were obtained using a Spectrum 65 FT-IR spectrometer (PerkinElmer Co., Ltd., Waltham, MA, USA) in the attenuated total reflection (ATR) mode.

The NMR spectra of EZT were obtained on a Bruker AVANCE III HD 400 spectrometer (Bruker BioSpin Corporation, Billerica, MA, USA) operating at 400 MHz. Deuterated chloroform was used as the solvent. Tetramethylsilane was used as an internal standard and as a reference for chemical shifts. Sixteen scans with 16 K data points were acquired for the EZT ^1^H NMR spectrum.

A Q10 differential scanning calorimeter (TA Instrument Co., New Castle, DE, USA) was used to analyze the thermal properties of the samples. The 5 mg sample was heated from 0 to 300 °C at a rate of 10 °C min^−1^ in a nitrogen atmosphere. Prior to measurement, all samples were completely dried to remove moisture.

The DMA of the samples was obtained with a Q800 dynamic mechanical analyzer (TA Instrument Co., New Castle, DE, USA) under the dual cantilever mode, as defined in ASTM D4065. The test sample dimensions were approximately 60.0 × 13.0 × 3.0 mm^3^. The sample was tested at 1 Hz and 3 °C min^−1^ from 30 to 140 °C. The maximum tan δ peak was recorded, which corresponded to the glass transition temperature of the sample.

The HDT of the samples was evaluated using a QM950H (Qmesys Co. Ltd., Anyang, South Korea) according to ASTM D648. The test sample dimensions were approximately 127.0 × 12.7 × 3.2 mm^3^. The sample was tested with a constant stress of 0.45 MPa by increasing the temperature at a rate of 3 °C min^−1^.

The thermal stability of the samples was evaluated using a TGA 4000 thermogravimetric analyzer (PerkinElmer Co., Waltham, MA, USA). The 10 mg sample was heated from 30 to 800 °C at a heating rate of 10 °C min^−1^ in a nitrogen atmosphere.

The miscibility and phase morphology of the samples were examined using a Quanta FEG 250 scanning electron microscope (FEI Co., Ltd., Salem, OR, USA). The sample was cryo-fractured under liquid nitrogen, and the resulting fractured sample was coated with a thin layer of platinum (Pt) prior to examination at the ambient temperature.

The X-ray diffraction patterns of the samples were collected and analyzed using an automated multipurpose X-ray diffractometer (SmartLab 9 kW system, Rigaku Co., Tokyo, Japan) with Cu Kα (λ = 1.54 Å) radiation at 40 kV and a 40 mA electric current for 2θ angles of 10–90°.

The tensile strength and Young’s modulus of the samples were measured using a QM-100 T universal testing machine (Qmesys Co. Ltd., Anyang, South Korea). Five specimens were prepared using an LGE-110 II electric injection molding machine (LS Mtron Co., Ltd., Anyang, South Korea) according to the ASTM D 638-14 standard for dumbbell-shaped samples, type 1. The transparency of the samples was measured using a UV-vis spectrophotometer (Shimadzu—UV 2600, Tokyo, Japan) in the diffuse reflectance spectroscopy transmission mode.

## 3. Results and Discussion

### 3.1. Chemical Structures

**Figure 2:**
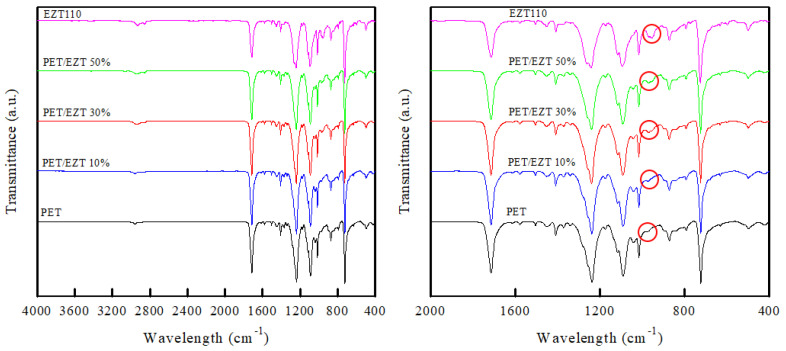
ATR-FTIR spectra of the polyethylene terephthalate (PET)/ECOZEN^®^T110 (EZT) samples.

ATR-FTIR spectroscopy was performed to determine the molecular interactions between PET and EZT in the PET/EZT samples. As shown in Figure 2, the characteristic bands of the pristine PET were observed at 2956 and 2867 cm^−1^ (C–H stretching of the CH_2_ groups), 1712 cm^−1^ (C=O stretching of carboxylic ester group), 1576, 1509, and 1409 cm^−1^ (aromatic skeleton stretching), 1453 and 873 cm^−1^ (CH_2_ bending and CH_2_ rocking), 1238 and 1089 cm^−1^ (C–C–O stretching of ester group), 1015 and 725 cm^−1^(in-plane C–H stretching and out-of-plane C–H bending of aromatic ring) [18]. The PET/EZT samples exhibited absorption bands relatively similar to that of pristine PET. However, weak doublet peaks at 973 (C–O stretching of ethylene glycol) and 958 cm^−1^ (C–H stretching of cyclohexylene ring) were observed, and the peaks’ intensity increased with increasing EZT content [19,20]. However, a new peak or peak shift was not observed, which implies that PET and EZT may have a weak interaction.

To determine the chemical structure of EZT, H^1^ Nuclear magnetic resonance (NMR) spectroscopy was performed. As shown in Figure S1, the peak at a chemical shift (δ) of 4.72 ppm was assigned to the hydrogen atom (HA) 1 of the ethylene glycol (EG) moiety, which overlapped with HA 8 of the ISB moiety. The peaks at δ 4.33, δ 5.52, δ 5.11, δ 4.72, δ 5.47, δ 4.22, and δ 4.11 ppm were attributed to the HA 5, 6, 7, 8, 9, 10a, and 10b of the ISB moiety, respectively. Peaks 5 and 10a overlapped with HA 11 cis and 11 trans of the CHDM moiety, respectively. The peaks at δ 4.22, δ 4.13, δ 1.84, δ 2.09, δ 1.97, δ 1.68, δ 1.19, and δ 1.61 were assigned to HA 11 trans, 11 cis, 12 trans, 12 cis, 13a trans, 13a cis, 13b trans, and 13b cis, respectively [17]. The ring structure of the CHDM can flip via a twisted-boat structure, leading to great chain mobility for EZT [21]. In addition, ISB has two tetrahydrofuran rings, which is a unique rigid molecular structure corresponding to a high T_g_ of EZT [16,17].

### 3.2. Thermal Properties and Thermal Stability

**Figure 3:**
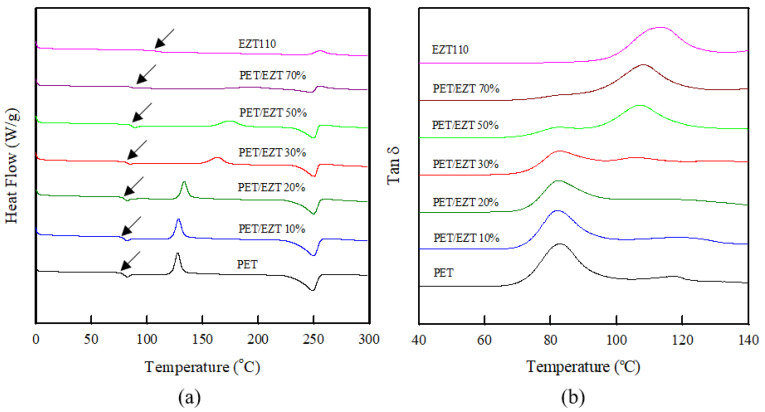
(**a**) DSC curves and (**b**) tan δ curves of the PET/EZT samples.

In general, T_g_ is a vital parameter because profound changes in the physical properties of the polymers, such as the thermal expansion coefficient and modulus, occur at this temperature [22]. The existence of a single T_g_ is proof of miscibility in binary systems (i.e., polymeric systems). In addition, the shift of T_g_ relative to the T_g_ values of the component polymers demonstrates the degree of phase separation [23]. The DSC thermograms of the PET/EZT samples are shown in Figure 3a, and their thermal properties are summarized in Table 1. The T_g_ values of the pristine PET and EZT were 79.0 °C and 107.8 °C, respectively. The T_g_ of ECOZEN^®^ T110 was higher than that of ECOZEN^®^ HF502 (in a previous study) [16] because of the high ratio of ISB (rigid molecular structure) in EZT [21]. With increasing EZT content, the T_g_ of the PET phase shifted to higher temperatures. This indicated that the dimensional stability of the PET/EZT samples was improved. A single T_g_ was observed for the PET/EZT samples with low EZT contents (10–20%), which implied miscibility in these compositions. However, two T_g_ values were obtained by adding EZT contents over 20%, the T_g_ of the PET phase shifted to a higher temperature, and the T_g_ of the EZT phase shifted to a lower temperature. This implies that the PET/EZT samples with high EZT contents (30–70%) are partially miscible [24,25]. In general, transesterification reaction can occur between two polymers during melt blending, which depends on blending conditions (e.g., temperature, duration of mixing, and the presence of transesterification catalyst) [26]. Several research groups reported that the transesterification reaction is usually occurred near and above the melting temperature of the polymer, which has also been observed in the polyester blends (e.g., PET/PEN, PET/PC, etc.) [26,27,28]. This chemical reaction can affect the miscibility of the sample by increasing the degree of miscibility through the formation of copolymers [26,29,30,31]. In this study, the melt blending was performed near and above the melting temperature of PET, therefore, the presence of miscibility and partial miscibility may be related to the transesterification reaction as reflected by the presence of single T_g_ and shifting of T_g_ towards the intermediate value between those of two polymers [27,32].

The pristine PET sample exhibited a melting temperature (T_m_) and enthalpy of fusion (ΔH_m_) of 249.3 °C and 38.2 J g^−1^, respectively. However, the pristine EZT sample did not show T_m_ and ΔH_m_, which was related to its completely amorphous structure. With increasing EZT content, a slight decrease in the T_m_ and a remarkable decrease in the ΔH_m_ of the PET/EZT samples were observed because crystal growth was inhibited by the amorphous polymer [33]. The reduction in T_m_ and crystallinity as reflected by ΔH_m_ could be related to the effect of transesterification reaction during melt blending [34,35]. This result was consistent with the measurement by Zheng et al. [35], which reported that the crystallinity and T_m_ of the PET in the PC/PET samples decreased due to the transesterification reaction during melt-processing. The pristine PET showed a cold-crystalline temperature (T_cc_) of 127.6 °C and enthalpy of cold-crystalline (ΔH_cc_) of 27.2 J g^−1^, respectively. The appearance of T_cc_ during the heating scan in DSC analysis was attributed to the polymer segments acquiring the necessary mobility to rearrange into crystalline domains [36]. In contrast, the pristine EZT did not exhibit T_cc_ or ΔH_cc_, indicating a completely amorphous polymer. Notably, the cold-crystalline peak broadened and shifted toward a higher temperature, and the ΔH_cc_ of the PET/EZT samples decreased with increasing EZT content. This demonstrates a low crystallization ability owing to the presence of an amorphous phase from EZT [37], which could be related to the effect of the transesterification reaction. This result was consistent with the investigation by Na et al. [34], indicating that the occurrence of transesterification between poly(trimethylene terephthalate) and polycarbonate resulted in a reduction in T_m_, crystallinity, and ΔH_cc_ while induced an increase in the T_cc_ of the samples.

Dynamic mechanical analysis was performed to detect the second-order transition or glass transition temperature of the polymeric systems [38]. The tan δ peak corresponding to the T_g_ of pristine PET, PET/EZ, and EZT samples is illustrated in Figure 3b and summarized in Table 1. The pristine PET and pristine EZT exhibited individual T_g_ values of 83.0 °C and 112.7 °C, respectively. Similar to the DSC results, only PET/EZT samples with low EZT contents showed a single T_g._ This indicated that miscible products could be obtained by adding an EZT ≤ of 20%. However, two T_g_ values were observed for the PET/EZT samples with high contents (30–70%). The T_g_ of the PET phase remained stable, and the T_g_ of the EZT phase shifted to a higher temperature. This also implied that the PET/EZT samples were partially miscible [25,39]. However, further evidence for supporting the partial system can be determined based on the transparency of the samples.

**Figure 4:**
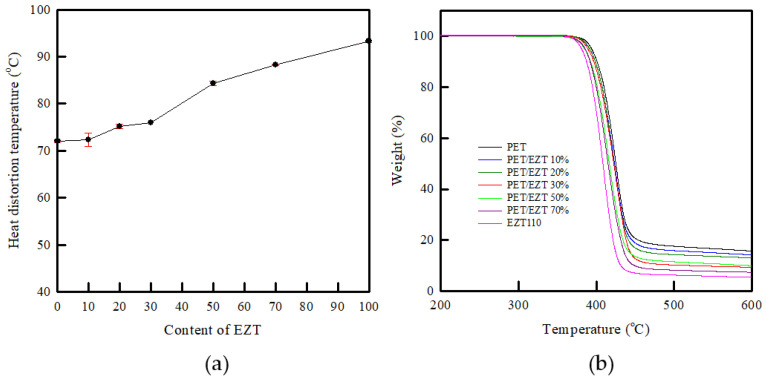
(**a**) HDT and (**b**) TGA curves of the PET/EZT samples.

High-performance materials mostly utilize high HDTs to maintain their mechanical properties at high temperatures. The HDT of the materials strongly depends on the glass transition temperature, hardness, and stiffness [40,41]. The effects of EZT on the changes in HDT of the PET are shown in Figure 4a and Table 2. The pristine PET exhibited an HDT at 72.1 °C, whereas the pristine EZT was 93.3 °C. Evidently, the HDT of pristine EZT was higher than that of pristine PET owing to the rigid molecular structure of ISB in the EZT. As expected, the HDT of the PET/EZT samples apparently increased with increasing EZT content, from 72.1 °C (pristine PET) to 88.3 °C (PET/EZT 70%). This change may arise from the chain mobility of PET being restricted by the high number of rigid ISB groups in EZT, resulting in enhanced resistance to thermal deformation under load. Notably, an increase in the HDT value is consistent with an increased T_g_ value in the DSC analysis. This confirms that the dimensional stability of the PET is improved by the introduction of EZT, although some samples are partially miscible. The partial miscibility between PET and EZT could be attributed to the high concentration of bulky ISB and CHDM groups in EZT.

In general, the thermal stability of polymeric systems strongly depends on the chemical structure, molecular weight, and degree of crystallinity [42,43]. The thermal stability of the PET/EZT samples is shown in Figure 4b, and the decomposition temperatures are summarized in Table 2. Pristine PET exhibited a degradation temperature at 10% weight loss (T_d10%_) of 401.8 °C, whereas EZT presented T_d10%_ at 387.5 °C. This indicated that the thermal stability of PET was higher than that of EZT, which was related to its semi-crystalline structure [43]. In the PET/EZT samples, as the EZT content increased, a lower degradation temperature and residue of the PET/EZT samples were observed. This demonstrated a reduction in the thermal stability of the samples owing to a reduction in crystallinity (an increase in free volume) that was affected by the addition of the amorphous EZT and the partial miscibility of the samples; this enabled faster thermal degradation via chain scission of both polymers. However, all the PET/EZT samples exhibited a one-step decomposition, which implied that the introduction of ECOZEN T110 did not affect the original decomposition pattern of pristine PET. Although the thermal stability was reduced from 401.8 °C to 391.8 °C, the conventional food and beverage packaging materials were mostly subjected to temperatures below such a temperature during the sterilization or reheating processes [44,45,46]. Therefore, there is no limitation in using the PET/EZT systems instead of the pristine PET as a packaging material.

### 3.3. Morphologies

**Figure 5:**
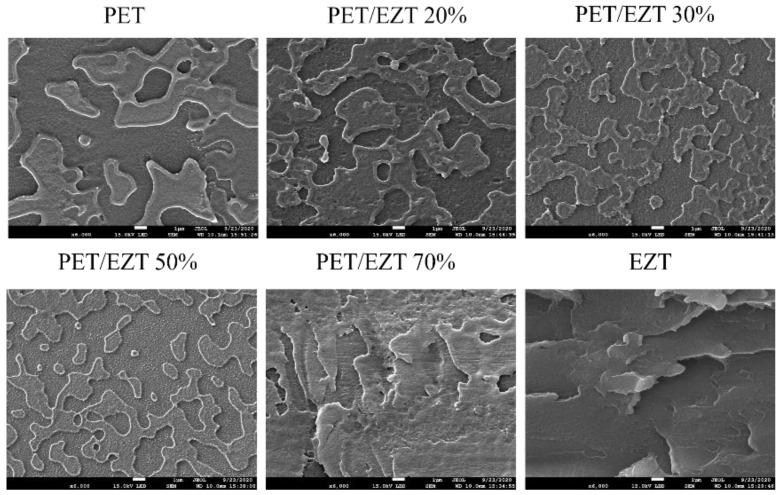
SEM micrographs of the cryo-fractured surface of PET/EZT samples.

Generally, a miscible polymeric system is identified as a homogeneous structure at the molecular level, while the lower range of homogeneous structure (limited) is observed in the partially miscible polymeric system, in which the separation phase can occur with the domination of one of the blend component. In addition, an immiscible polymeric system results in a heterogeneous morphology with apparently separated phases, including pristine components [47]. Scanning electron microscopy (SEM) was employed to further evaluate the miscibility and morphology. The SEM micrographs of the cryo-fractured surfaces of the PET/EZT samples are shown in Figure 5. The pristine PET exhibited a smooth surface for both convex and flat regions, and these different regions may be attributed to its semi-crystalline structure. The pristine EZT showed a smooth surface and continuous phase that corresponded to its fully amorphous structure. At a relatively low content of EZT (20%), the phase morphology of the PET/EZT samples was similar to that of the pristine PET, despite a slightly rough surface. This may imply the miscibility between PET and EZT. By increasing the content of EZT up to 50%, the surface became rougher, and the size of the convex region became smaller. At a relatively high EZT content (70%), the PET/EZT sample had a rough surface in which the phase morphology was relatively similar to that of the pristine EZT. This could be related to an EZT-rich phase. Based on the SEM results, the PET/EZT samples with high EZT contents (>20%) are partially miscible, which is consistent with the DSC and DMA results.

**Figure 6:**
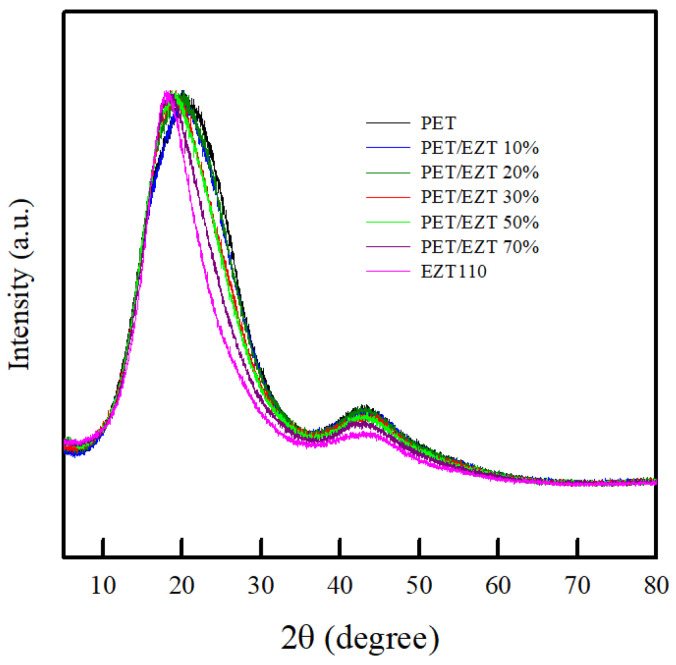
XRD patterns of the PET/EZT samples.

XRD analysis was performed to identify the crystalline structure of materials. Generally, crystalline polymers represent a series of sharp peaks in the XRD patterns, and amorphous polymers exhibit a relatively broad halo. In contrast, semi-crystalline polymers show a superposition of crystalline reflections on an amorphous halo [48]. The XRD patterns of pristine PET, pristine EZT, and PET/EZT samples are shown in Figure 6. The pristine PET sample exhibited a characteristic broad peak and a weak peak at a diffraction angle (2θ) of 20.53° and 43.16°, which corresponded to the amorphous phase of the unstretched PET sheet. This may have been related to a rapid cooling rate during the sheet forming process (injection molding). Therefore, long molecule chains did not have sufficient time to rearrange to form a crystalline phase at low temperatures. The pristine EZT sample also exhibited a broad halo corresponding to its completely amorphous structure. All PET/EZT samples showed a diffraction pattern similar to that of the pristine PET and pristine EZT samples. This indicates that the introduction of EZT into PET does not change the XRD pattern of PET. However, as the content of EZT increased, the characteristic peak shifted slightly to a low diffraction angle. This implies that the interlayer spacing of the PET chain slightly increased (lose of crystallinity), which could be related to transesterification reaction [31,34].

### 3.4. Mechanical Properties

The mechanical properties of polymeric systems, such as tensile strength and Young’s modulus, rely on the composition, level of compatibility, and degree of crystallinity. The mechanical properties of the polymeric systems can be improved when the two components are compatible owing to strong interfacial adhesion [49]. As shown in Table 3, pristine PET exhibited higher tensile strength and Young’s modulus than pristine EZT. This is attributed to a high degree of crystallinity in the semi-crystalline structure of PET. This crystallinity results in harder and stiffer behaviors that differ from EZT, with a softer behavior corresponding to its amorphous structure. As the content of EZT increased, the tensile strength and Young’s modulus of the PET/EZT samples were slightly reduced. These changes were related to a reduction in crystallinity (synergistic effect) affected by the addition of the amorphous EZT in the samples. Consequently, the PET/EZT samples became softer and resulted in a decrease in yield stress, as shown in the stress–strain curve (Figure S2). In addition, an increase in the toughness of the PET/EZT samples was observed, as reflected by the area of the stress–strain curves. This is attributed to the higher content of the ductile CHDM group in EZT [50].

### 3.5. Transparency

**Figure 7:**
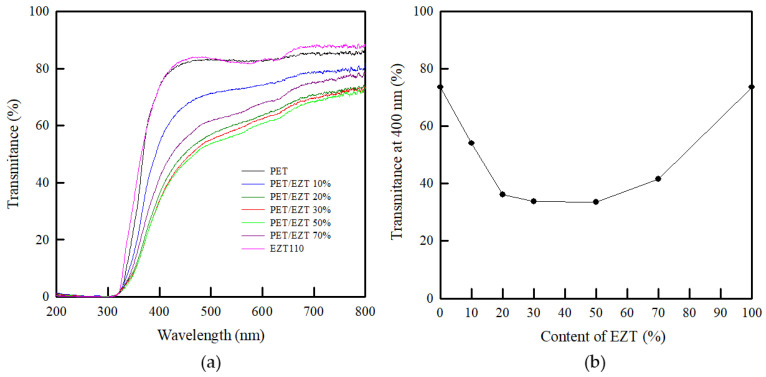
(**a**) UV-vis curves and (**b**) transmittance at 400 nm of the PET/EZT samples.

Transparency is an essential property of primary packaging (e.g., bottles, trays, and lid films) to show a packaged product. Highly transparent polymeric materials can be used instead of glass, which reduces the cost of shipping owing to its lightweight. In general, polymeric systems are transparent if the transparent components in the system are miscible at the molecular level; otherwise, the systems tend to be opaque. However, the polymeric systems can present a transparent property despite being immiscible if the difference in the refractive index for the blending components is lower than 0.01 [51]. The transparency of PET/EZT samples can be investigated using UV-vis diffuse reflectance spectroscopy. As shown in Figure 7, pristine PET and pristine EZT showed high transmittance owing to the amorphous phase of their structures. However, the PET/EZT samples exhibited a reduction in transparency depending on the EZT content. The transparency of the PET/EZT samples decreased when the weight ratio of the components in the blend was approximately equal (PET/EZT: 50/50). Moreover, the transparency of the PET/EZT samples with high EZT contents was apparently lower than that of pristine PET, thus implying partial miscibility between PET and EZT; this was consistent with the SEM and DSC results. In a previous study, PET/PEICT samples exhibited high transparency, which was similar to that of pristine PET owing to the presence of truly miscible blends. In this study, however, partial miscibility between PET and EZT was observed, which may be attributed to slightly different rheological and thermodynamic parameters being affected by a higher ratio of bulky (rigid ISB and ductile CHDM) groups in EZT [52,53].

## 4. Conclusions

An amorphous bio-based terpolyester (EZT) with a high ratio of rigid ISB and ductile CHDM groups was introduced into semi-crystalline PET to improve the dimensional stability of PET by the melt-extrusion method. The DSC and DMA results exhibited a single T_g_ for the PET/EZT samples with low EZT contents (≤20%), indicating the miscibility between PET and EZT in the samples. However, two T_g_ values were observed for PET/EZT samples with high EZT contents (30–70%), implying partial miscibility. This may have been caused by slightly different rheological and thermodynamic parameters affected by a higher ratio of bulky (rigid ISB and ductile CHDM) groups in EZT. As the content of EZT increased, the crystallinity of the PET phase in the PET/EZT samples decreased while the T_cc_ increased. Notably, the T_g_ and HDT of all PET/EZT samples increased, which demonstrated their enhanced dimensional stability. This change is related to the presence of an amorphous phase corresponding to the unique rigid molecular structure of the ISB group in EZT, which could be related to the transesterification reaction during melt blending. Finally, the as-prepared PET/EZT samples with improved dimensional stability can be used as a high-temperature polymeric material for various applications in the packaging, fiber, and automotive fields.

## Figures and Tables

**Table 1:** Thermal properties of the PET/EZT samples obtained from DSC and DMA analysis.

Samples	DSC						DMA	
	T_g1_ (°C) ^(a)^	T_g2_ (°C) ^(b)^	T_cc_ (°C) ^(c)^	ΔH_cc_ (°C) ^(d)^	T_m_ (°C) ^(c)^	ΔH_m_ (°C) ^(d)^	T_g1_ (°C) ^(a)^	T_g2_ (°C) ^(b)^
PET	79.0	-	127.6	27.2	249.3	38.2	83.0	-
PET/EZT 10%	79.7	-	128.3	27.7	250.0	35.0	82.3	-
PET/EZT 20%	80.4	-	133.4	20.7	250.7	30.0	82.1	-
PET/EZT 30%	83.0	102.6	164.3	18.0	250.8	20.0	82.7	107.1
PET/EZT 50%	87.1	105.2	175.8	17.7	249.8	18.2	81.4	107.1
PET/EZT 70%	90.6	106.9	194.8	6.7	248.5	10.2	81.9	108.4
EZT110	-	107.8	-	-	-	-	-	112.7

^(a)^ first observed glass transition temperature of the PET/EZT samples. ^(b)^ second observed glass transition temperature of the PET/EZT samples. ^(c)^ cold-crystalline temperature and melting temperature of the PET/EZT samples, respectively. ^(d)^ enthalpy of cold-crystalline and enthalpy of fusion of the PET/EZT samples, respectively.

**Table 2:** HDT and degradation temperature of the PET/EZT samples.

Samples	HDT (°C) ^(a)^	TGA	
		T_d10%_ (°C) ^(b)^	Residue (%) ^(c)^
PET	72	401.8	15.3
PET-EZT 10%	72	398.6	13.0
PET-EZT 20%	75	397.4	11.8
PET-EZT 30%	76	396.7	8.9
PET-EZT 50%	84	392.5	8.9
PET-EZT 70%	88	391.8	7.1
EZT110	93	387.5	4.8

^(a)^ HDT of PET/EZT samples. ^(b)^ Degradation temperature at 10% weight loss of PET/EZT samples. ^(c)^ Residue at 700 °C of PET/EZT samples.

**Table 3:** Tensile strength and Young’s modulus of the PET/EZT samples.

Samples	Tensile Strength (MPa)	Young Modulus (MPa)
PET	60.1 ± 0.2	2164.8 ± 6.8
PET/EZT 10%	59.6 ± 0.1	2125.3 ± 18.9
PET/EZT 20%	58.1 ± 0.2	2069.0 ± 13.8
PET/EZT 30%	57.7 ± 0.2	2043.9 ± 14.0
PET/EZT 50%	55.7 ± 0.1	1967.4 ± 8.0
PET/EZT 70%	53.5 ± 0.2	1875.1 ± 15.7
EZT110	49.2 ± 0.1	1723.2 ± 7.0

## Data Availability

The data presented in this study are available on request from the corresponding author.

## Supplementary Materials

The following are available online at https://www.mdpi.com/2073-4360/13/5/728/s1, Figure S1:^1^H NMR spectrum of EZT, Figure S2: Stress–strain curve of the pristine PET, PET/EZT samples, and pristine EZT.
