# Supplementary material for: PET/Bio-Based Terpolyester Blends with High Dimensional Thermal Stability
Source: Polymers (Basel). 2021 Feb 27;13(5):728. doi: 10.3390/polym13050728 (PMC7956836; doi:10.3390/polym13050728)
Supplement: Supplementary file 1 [file polymers-13-00728-s001.pdf]

Supplementary Materials

PET/bio-based terpolyester blends with high dimensional thermal stability

Sangyoon Park†, Sarinthip Thanakkasaranee†, Hojun Shin†, Youngsoo Lee, Guman Tak, Jongchul Seo*

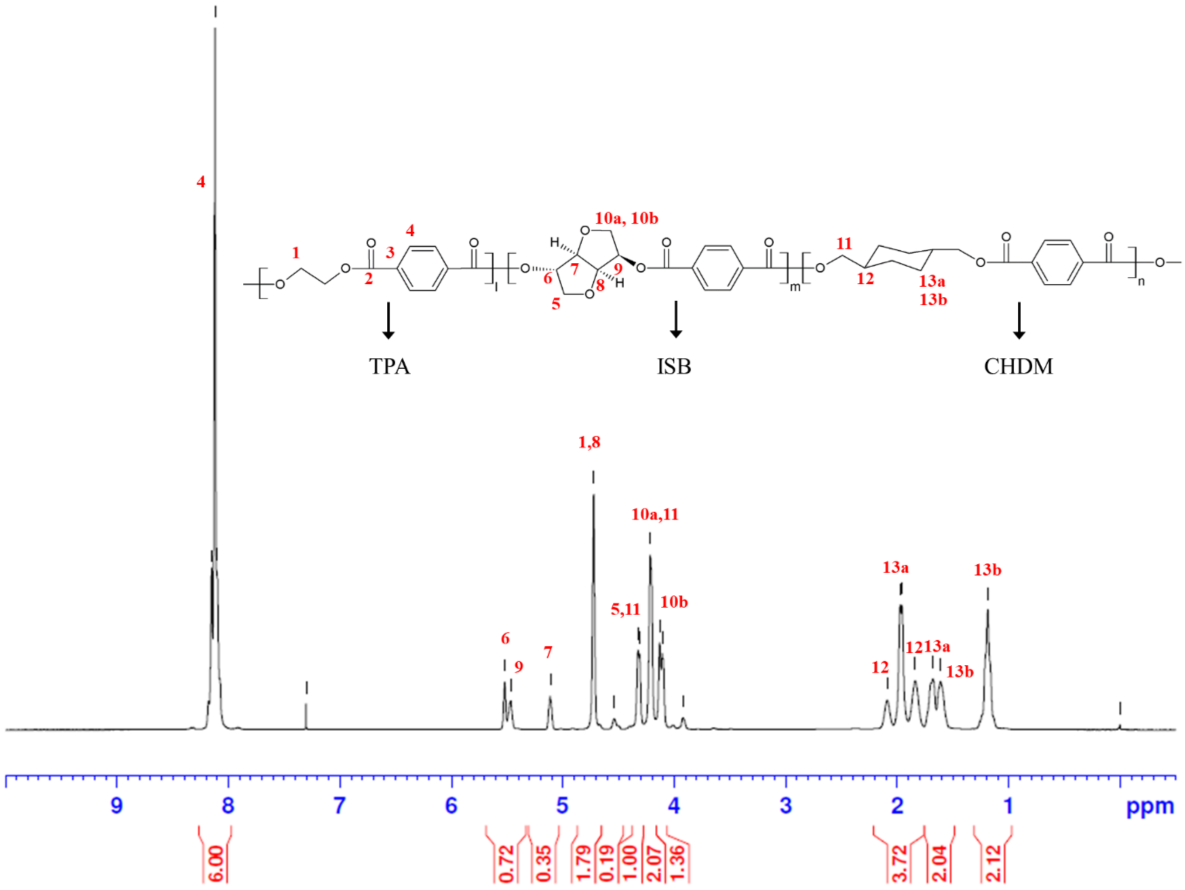

Figure S1. ^{1}H NMR spectrum of EZT.

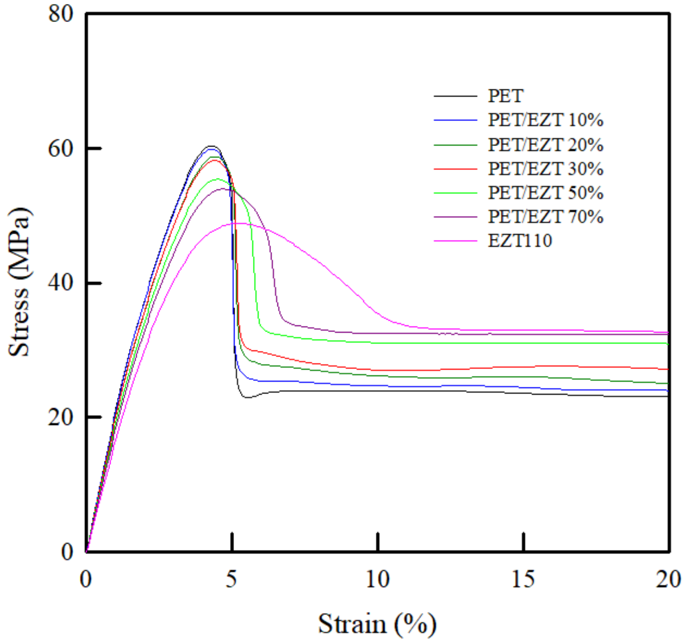

Figure S2. Stress–strain curves of the pristine PET, PET/EZT samples, and pristine EZT.
